# Immunological Analysis of Oral Cytobrush Specimens for Early Detection of Oral Cancer Biomarkers: A Comprehensive Review

**DOI:** 10.3390/ijms27042059

**Published:** 2026-02-23

**Authors:** Reem Hanna, Alberto Luigi Rebaudi, Saman Warnakulasuriya, Senada Koljenovic, Maria Menini, Francesco Laganà, Bernardo Bianchi, Paolo Iacoviello, Mauro Labanca, Marco Greppi, Federico Rebaudi, Silvia Pesce, Alberto Rebaudi, Emanuela Marcenaro

**Affiliations:** 1 Department of Restorative Dental Sciences, Eastman Dental Institute, Medical Faculty, University College London, London WC1E 6DE, UK; 2 Head and Neck Academic Centre, Integrated Research, Division of Surgery and Interventional Science, Medical Faculty, University College London, London W1W 7TY, UK; 3 Dental Faculty, Royal College of Surgeons Ireland, 21–122 St Stephen’s Green, Dublin 2, D02 H903 Dublin, Ireland; 4 Department of Surgical Sciences and Integrated Diagnostics, University of Genoa, Viale Benedetto XV, 6, 16132 Genoa, Italy; 5 Private Dental Practice, Piazza della Vittoria 8/12, 16121 Genoa, Italy; 6 Faculty of Dentistry, Oral & Craniofacial Sciences, King’s College London, London SE1 9SP, UK; 7 Department of Pathology, Antwerp University Hospital, 2650 Antwerp, Belgium; 8 Department of Maxillofacial Surgery, Unità Operativa Complessa di Chirugia Maxillofaciale Odontoiatra, IRCCS Ospedale San Martino, Largo Rosanna Benzi, 10, 16132 Genoa, Italy; 9 Department of Maxillofacial Surgery, Galliera Hospital, Via Mura delle Cappuccine 14, 16128 Genoa, Italy; 10 Department of Anatomy and Physiopathology, University of Brescia, Viale Europa 11, 25123 Brescia, Italy; 11 Department of Experimental Medicine (DIMES), University of Genoa, Via Leon Battista Alberti 2, 16132 Genoa, Italy; 12 IRCCS Ospedale Policlinico San Martino, Largo Rosanna Benzi, 10, 16132 Genoa, Italy

**Keywords:** cytokines, early detection, immunological biomarkers, oral cytobrush, oral potentially malignant disorders, oral squamous cell carcinoma, precision medicine, tumour suppressor proteins

## Abstract

Early identification of the risk of malignant transformation in oral potentially malignant disorders (OPMDs) is critical for improving outcomes in oral squamous cell carcinoma (OSCC). This comprehensive review examines immunological biomarkers obtained from minimally invasive oral cytobrush (OCB) specimens for the early detection of OSCC within a precision medicine framework. The objectives were to (1) identify and characterise key immunological biomarkers associated with early oral carcinogenesis; (2) evaluate the diagnostic utility of OCB sampling for detecting these biomarkers; and (3) explore the potential of OCB-based profiling to support personalised screening and patient management. The review highlights the potential advantages of OCB compared with conventional diagnostic methods, as reported in the literature, particularly its ability to capture early malignant changes through immunological analysis. Evidence is discussed for biomarker pathways related to cell-cycle and differentiation dysregulation (p53, Ki-67, CKs), inflammation-driven epithelial transformation (IL-1β, IL-6, IL-8, TNF-α), and immune suppression and checkpoint activation (PD-L1, B7-H6). OCB provides reliable and patient-friendly cyto-salivary samples that are suitable for immunological and molecular analyses. Aberrant biomarker expression detected in OCB specimens correlates with epithelial dysplasia and reflects early non-invasive neoplastic transformation, supporting the diagnostic value of integrated biomarker panels. Overall, OCB-based immunoanalysis represents a practical, non-invasive approach for the early detection of OSCC. Emerging technologies, including AI and multi-omics approaches, may further support the precision and predictive values of immunological analysis for OSCC. When combined with relevant biomarker pathways reflecting tumour biology and host immune responses, this strategy could offer a strong foundation for precision-medicine screening. It may also support personalised monitoring in patients with OPMDs.

## 1. Introduction

Oral cancer (OC) remains a major global public health challenge. Oral squamous cell carcinoma (OSCC) accounts for more than 90% of all OC cases, and is among the most common malignancies worldwide [1]. Despite advances in treatment, OSCC continues to have a poor prognosis, with an average overall five-year survival rate of approximately 50% [2]. Importantly, survival outcomes differ markedly by stage at diagnosis, with five-year survival rates exceeding 80–90% for stage I disease, but falling to below 30–40% in advanced-stage OSCC [3]. This striking survival disparity underscores the critical importance of early detection strategies capable of identifying malignant transformation at preclinical or early-stage disease.

Notably, nearly 70% of OSCC cases are diagnosed at advanced stages, largely because early lesions are often asymptomatic and clinically subtle, resulting in significant patient- and professional-related diagnostic delays [4,5,6]. Additionally, it exhibits biological and clinical characteristics distinct from those of many other solid tumours. This can often lead to delayed diagnosis and rapid progression from potentially malignant lesions to invasive carcinoma. In some cases, this progression can occur with limited or undetectable intermediate dysplastic changes [5,6]. This diagnostic delay is further compounded by the subtle nature of early epithelial alterations and the anatomical complexity of the oral cavity. Additionally, the limitations of conventional visual and tactile examination in routine clinical practice contribute to this challenge [7,8]. Consequently, current OSCC screening methods remain operator-intensive for the early detection of OSCC and oral potentially malignant disorders (OPMDs), especially at preclinical or early disease stages [7,8]. Therefore, there is a strong need for reliable and minimally invasive screening methods that can detect oral lesions at their earliest and most treatable stages.

### 1.1. Limitations of Conventional Diagnostic Biopsy Methods

Histopathological examination of tissue obtained via oral biopsy remains the diagnostic “gold standard” for OSCC [5]. However, this approach has several important drawbacks, which are listed below:

- Invasiveness: histological diagnosis requires a tissue biopsy, an inherently invasive procedure. Although generally safe, it can cause discomfort, and many necessitate additional follow-up procedures, often resulting in lower patient compliance. This is particularly relevant in OC screening, where early detection may require multiple biopsies from different sites. Minimally invasive alternatives, such as OCB sampling, offer a patient-friendly approach that reduces procedural burden while enabling repeated collection for diagnostic purposes [9].
- Sampling Error: the reliability of histopathology is highly dependent on the quality/representativeness of the biopsy sample. Oral lesions can be heterogeneous, and if the biopsy does not capture the most representative tissue, or is taken from a less affected area, it may fail to detect malignancy or dysplasia [10]. This is particularly problematic in cases where cancer is localised or at an early stage, where lesions might be large in extent or difficult to access [11].
- Inter-Observer Variability: histopathological interpretation can vary between pathologists, particularly when evaluating subtle architectural and cytological changes. This variability is most pronounced in the assessment and grading of oral epithelial dysplasia and OPMDs, where diagnostic thresholds are less well defined [11]. Although histopathology remains essential for the diagnosis of OSCC, differences in interpretation may influence diagnostic categorisation and grading, potentially affecting clinical decision-making.
- Limited Ability to Detect Early-Stage Cancer: histopathology often relies on the presence of visible lesions that can be biopsied. However, early-stage OSCC or potentially malignant lesions may not present obvious signs, leading to delayed diagnosis with, as a consequence, missed opportunity for successful early treatment [10]. By the time a diagnosis can be proved based on the representative biopsy, cancer may already have progressed, reducing the chances of successful treatment.
- Tissue Preservation and Processing Issues: the reliability of histopathology is contingent upon proper tissue preservation and processing. Delays in fixation or suboptimal tissue handling can lead to degradation, making it difficult to accurately assess the sample [12]. Even small errors in tissue preparation can lead to diagnostic inaccuracies, particularly when diagnosing early-stage lesions that require precise evaluation.
- Incomplete Molecular and Genetic Information: histopathology primarily provides information on tissue cellular morphology, but it does not capture molecular or genetic alterations that can be critical for personalised cancer treatment. Many OSCCs exhibit distinct genetic mutations or molecular changes (e.g., TP53 mutations, EGFR alterations), which are not detectable via histopathological analysis alone [13]. In addition, histopathology is semi-quantitative, so it is difficult to measure the exact levels of specific biomarkers [14]. Tissue processing may also change the tissue and reduce information about its original state [14]. Several studies have shown that single biomarkers or biomarker panels can help with the early detection of OSCC and pre-malignant lesions [15,16,17,18,19,20,21].
- Ability to Detect Micrometastasis or Regional Spread: histopathology is effective in diagnosing localised tumours, but it is less reliable in detecting micrometastasis or regional spread. Similarly, OCB sampling is limited to local epithelial assessment, and cannot detect metastatic dissemination. Early cancer metastasis often occurs before clinical signs are visible, and neither histopathology nor OCB may identify small tumour deposits in distant tissues or lymph nodes, requiring additional imaging techniques [22,23].

### 1.2. Oral Cytobrush—Minimally Invasion Method

The OCB is a minimally invasive technique used to collect squamous epithelial cells from the oral mucosa for microscopic analysis. It serves as an adjunctive screening tool for detecting cellular atypia and early malignant changes without the need for surgical biopsy, making it well suited for repeated patient-friendly sampling [6,7]. Due to its ease of use, tolerability, and applicability for longitudinal monitoring, OCB-based sampling aligns very well with modern screening and precision medicine paradigms, particularly in high-risk populations or in settings where invasive biopsy may be unfeasible.

The OCB provides clinicians with a non-invasive diagnostic tool analogous to the “Pap Smear Test” used for early cervical cancer detection through OCB sampling. This approach has been shown to significantly reduce morbidity rates by over 80%, while minimising the need for extensive treatments [2].

In the oral and maxillofacial surgery field, OCB has been proposed as a chairside diagnostic screening tool enabling the simultaneous harvesting of target tissue cells or the collection of unstimulated saliva [24]. Consequently, it can be applied for OPMDs for the early detection of dysplastic changes in asymptomatic patients or those presenting with mild symptoms, in cases where an immediate biopsy may not be justified.

The diagnostic role of OCB in evaluating suspicious oral lesions compared to the standard surgical incisional biopsy has been debated, due to the limited availability of clinical data [25]. Nevertheless, several studies have demonstrated OCB effectiveness in harvesting cyto-salivary samples for molecular biomarker analysis and for the early diagnosis of OSCC [26,27,28,29,30].

In a preliminary comparative clinical study, Rebaudi et al., 2023 [29] evaluated the performance of OCB in detecting tumour-associated biomarkers in patients with OSCC.

Using an automated ELISA technique for immunological approach, the authors demonstrated that OCB-derived samples could distinguish between tumour tissue and non-tumour oral epithelium The results closely aligned with those from gold-standard surgical biopsy and immunohistochemical analyses. Similarly, Gissi et al., 2021 [28] validated the use of OCB for the detecting early oral dysplasia through genetic biomarker analysis. They showed that DNA methylation profiling of 13 genes in OCB samples can identify dysplastic changes associated with oral carcinogenesis.

In a prospective blinded study conducted by Nanayakkara et al., 2016 [31], the authors compared cytological sampling using OCB and a conventional spatula with histopathological findings. The authors reported that OCB-based sampling showed favourable diagnostic performance compared with spatula-based cytology. It also demonstrated good agreement with histopathology. These findings support its potential utility as a screening tool for the early detection of suspicious oral malignant and premalignant lesions.

In addition, Aro et al., 2019 [27] demonstrated that OCB can effectively harvest OSCC-related molecular markers. It can also simultaneously collect unstimulated saliva samples from patients with head and neck cancer (HNC). These findings support OCB’s potential as a non-invasive diagnostic tool. However, the authors emphasised that no single biomarker is sufficient for detecting early disease onset. Therefore, using multiple biomarkers is necessary to improve diagnostic accuracy and sensitivity in OSCC detection [32]. Overall, OCB can be considered a minimally invasive and reproducible alternative for diagnostic and/or screening purposes in patients with OPMDs [26].

### 1.3. Importance of Immunological Biomarkers for Early Detection of OSCC

Immunological biomarkers can assist in the early detection of OSCC. They reflect molecular and cellular changes that occur before visible malignant changes in tissue. Some biomarkers indicate early dysplasia or neoplastic transformation. These include tumour-suppressor proteins like p53 and proliferation markers such as Ki-67. Other biomarkers are linked to malignant signalling, including growth-factor receptors like EGFR. Inflammatory cytokines such as IL-1β, IL-6, IL-8, and TNF-α reflect changes in the tumour microenvironment and immune activation. Immune-checkpoint molecules, including PD-L1 and B7-H6 may indicate early immune evasion [8,9,32].

Incorporating these immunological markers in cytobrush-derived specimens may allow non-invasive, repeatable screening, guiding oriented monitoring and early intervention, and therefore advancing the goals of precision medicine in oral oncology and HNC.

To improve transparency and clarify the scope of this comprehensive review, the literature was identified through targeted searches of major biomedical databases, primarily PubMed, Scopus, and Web of Science. The review focuses on studies published mainly over the past two decades that investigated immunological and molecular biomarkers in OCB specimens, dysplastic lesions, or OSCC tissue, with an emphasis on clinical, translational, and experimental research relevant to early detection and precision medicine. Additional relevant publications were identified through manual screening of reference lists of key articles. Based on this comprehensive literature coverage, the aim of this review was to examine immunological biomarkers obtained from minimally invasive OCB specimens for the early detection of OSCC within a precision medicine framework. The objectives were to:

- Identify and characterise key immunological biomarkers linked to early oral carcinogenesis.
- Evaluate the diagnostic utility of minimally invasive OCB sampling for detecting these biomarkers.

Assessing cytobrush-based biomarker profiling can be integrated into a precision-medicine approach for early screening and individualised patient management.

## 2. Immune/Biomarker Pathway

### 2.1. Tumour Suppressor Pathways

The control of cell cycle progression, DNA repair mechanisms and epithelial differentiation are foundational in preventing malignant transformation of the oral mucosa.

One of the most studied tumour suppressors is p53, often described as a “guardian” of the genome. It halts the cell cycle in response to DNA damage, activates DNA repair mechanisms, and triggers apoptosis when the damage is irreparable [33]. Loss of p53 function or aberrant accumulation of mutant p53 is frequently observed in early oral epithelial dysplasia and overt OSCC [33].

For example, immunohistochemical studies show that the percentage of p53-positive cells increases markedly when comparing normal oral mucosa to dysplastic lesions and to OSCC (15–25% in normal, up to ~95% in malignant mucosa) in one series [34]. However, standard immunostaining cannot reliably distinguish wild-type from mutant p53 protein, which limits interpretation of functional significance in immunoassays.

Complementing p53, the proliferation marker Ki-67 reflects the growth fraction of a given cell population. High Ki-67 labelling indices in dysplastic and malignant oral epithelial cells indicate increased proliferative activity, and correlate with higher-grade lesions and poorer prognostic parameters such as lymph-node metastasis [35].

Beyond these, epithelial differentiation can also be assessed via cytokeratin (CK) expression patterns. For instance, alterations in CKs such as CK17 in upper layers of dysplastic epithelium can distinguish dysplastic from normal epithelium in tongue lesions [36].

Together, these markers reflect key steps in malignant transformation. Loss of regulatory control is indicated by p53, increased cell proliferation by Ki-67, and altered epithelial differentiation by CKs. Incorporating these biomarkers into minimally invasive sampling methods (such as OCB) may offer a strategy for early molecular detection before clear morphological invasion occurs.

### 2.2. Inflammatory Pathway

Chronic inflammation is increasingly recognised as a facilitator of carcinogenesis, including in the oral mucosa. Pro-inflammatory cytokines, many regulated by the NF-κB pathway, create a microenvironment that supports genomic instability and epithelial proliferation. They also can contribute to angiogenesis and immune evasion.

For instance, the cytokines IL-6, IL-8 and TNF-α are significantly elevated in both tissue and saliva of patients with OSCC and in some OPMDs. A study found increased levels of IL-8 and TNF-α in saliva from OSCC samples compared with normal mucosa and OPMDs, as a result of measured immunohistochemistry. This suggests that IL-8, in particular, may be a sensitive marker of malignant transformation [37].

A systematic review and meta-analysis found that salivary IL-8, IL-6, TNF-α and IL-1β are significantly higher in OSCC patients compared with healthy controls. Among these biomarkers, TNF-α demonstrated the highest diagnostic accuracy, with sensitivity ~79% and specificity ~92% [38].

Mechanistically, IL-6 activates the JAK/STAT3 and MAPK/PI3K pathways, promoting epithelial proliferation, survival and angiogenesis. IL-8 contributes to neutrophil recruitment and angiogenesis, while TNF-α sustains NF-κB activation and up-regulation of anti-apoptotic genes [39].

In the context of OCB specimens, measuring these cytokines or their expression in harvested epithelial cells may serve as a minimally invasive marker of early inflammatory-mediated malignant change. This approach could provide a window into the tumour-promoting inflammation axis, and complements the proliferation and differentiation markers mentioned above.

Table 1 summarises representative biomarkers analysed using OCB and related specimens, together with their biological pathways and clinical relevance in oral epithelial dysplasia and OSCC. While some studies report preliminary diagnostic data, quantitative measures such as sensitivity and specificity are not consistently available. Hence, this table provides a qualitative overview, rather than a comparative efficacy analysis.

### 2.3. Immune Evasion and Checkpoint Markers: B7-H6, PD-L1

As malignant transformation progresses, tumour cells often evolve mechanisms to evade immune surveillance. B7-H6 is an emerging member of the B7 family of immune checkpoint ligands. It is not typically expressed in normal tissues, but is upregulated in OSCC. There, it can interact with natural killer (NK) cell receptor NKp30 and modulate anti-tumour immunity. A recent review highlighted B7-H6 as a promising immunotherapy target, given its tumour-restricted expression and its role in immune modulation [40].

PD-L1 is one of the most important immune checkpoint molecules. It binds to the PD-1 receptor on T cells and NK cells. This interaction reduces the activity of these cells and weakens anti-tumour immunity. PD-L1 expression can be induced in the tumour microenvironment and is a major mechanism by which tumours suppress the immune response [39]. Some human NK cells also express high levels of PD-1, leading to effects similar to those seen in T cells and further limiting immune control of tumours [41].

In OSCC, a systematic review of immune checkpoints found that B7-H6 and PD-L1 were among the seven checkpoint molecules associated with poor survival in at least one study [42].

From a precision medicine standpoint, detecting PD-L1 and B7-H6 (or their soluble forms) in samples taken with OCB can give early information about how lesions avoid the immune system [40,41]. This can help clinicians decide which patients need close monitoring or might benefit from immunotherapy or immune-based prevention.

## 3. Biomarkers for Early Oral Cancer Detection

Early oral carcinogenesis involves multiple molecular alterations affecting cell cycle regulation, epithelial differentiation, inflammation, and immune response. Detecting these changes through minimally invasive approaches such as OCB sampling could provide a promising diagnostic pathway for precision medicine in oral oncology. The key immunological biomarkers relevant to early detection are summarised below.

### 3.1. Tumour Suppressor/Proliferation Markers

The *TP53* gene encodes the p53 protein, a tumour suppressor regulating DNA repair, apoptosis, and genomic stability [43]. Mutations or overexpression of dysfunctional p53 protein represent some of the earliest molecular events in oral carcinogenesis [44,45].

Immunocytochemical studies demonstrate increased p53 expression in oral epithelial dysplasia and OSCC compared with normal mucosa. This suggests its potential utility as an early biomarker of malignant transformation [46,47].

More recently, Rebuadi et al., 2023 [29] demonstrated the feasibility of detecting OSCC-related biomarkers, including p53, using a non-invasive OCB-based approach in a cohort of 15 patients who were diagnosed with OSCC. The samples were analysed using a highly sensitive automatic ELISA technique, which allowed for the simultaneously (qualitative and quantitative) analysis of six biomarkers from a single OCB sample.

**Figure 1:**
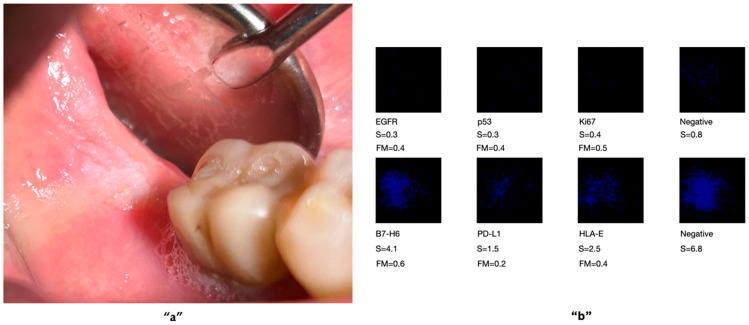
An immunological analysis was performed on an OCB sample obtained from a white lesion with a small ulceration in the retromolar region **“a”**. The results showed no cellular alterations, as none of the six tumour markers tested exhibited any expression **“b”**. However, given the lesion’s characteristics, the patient was enrolled in a periodic follow-up programme for ongoing monitoring. A biomarker is considered positive if the mean multiplication factor (FM) exceeds 1.1 and negative if the FM value is below 1.1. The test is deemed positive for a malignant tumour if the FM is >1.1 for all six biomarkers of interest [29]. Permission obtained from the authors.

**Figure 2:**
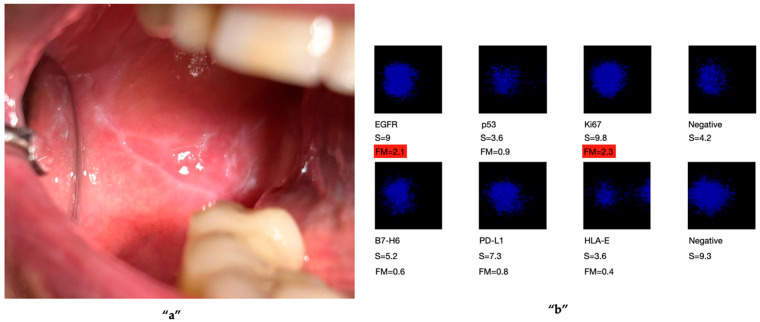
An immunological analysis was conducted on an OCB sample taken from an oral lichen planus lesion on the mucosa **“a”**. The results revealed cellular alterations, with two of the six tumour markers showing over-expression: EGFR (FM = 2.1) and Ki67 (FM = 2.3) **“b”**. Given the nature of the lesion, the patient was enrolled in a periodic follow-up programme for ongoing monitoring [29]. Permission obtained from the authors.

**Figure 3:**
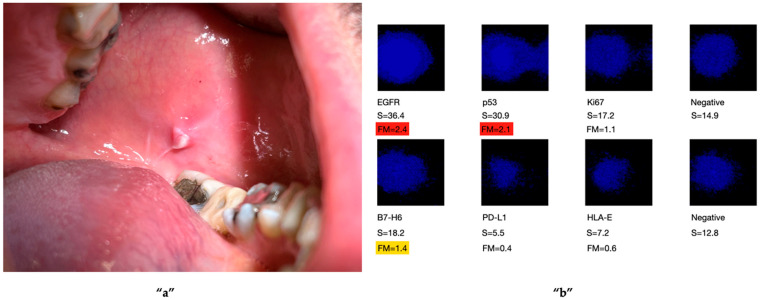
Immunological analysis of an OCB sample taken from a mucosal lesion **“a”**. The results revealed cellular alterations, with over-expression detected in three of the six tumour markers analysed [EGFR, p53, and B7-H6 **“b”**]. The lesion was surgically excised and histologically identified as an eroded traumatic fibroma of the oral mucosa. Given the nature of the lesion, the patient was enrolled in a periodic follow-up programme. A biomarker is considered positive if the mean multiplication factor (FM) exceeds 1.1 and negative if the FM value is below 1.1. FM values greater than 1.1 but less than 1.5 (indicating a moderate signal intensity) are highlighted in yellow, while values exceeding 1.5 (indicating a high signal intensity) are highlighted in red. The test is considered positive for a malignant tumour if the FM exceeds 1.1 for all six biomarkers of interest [29]. Permission obtained from the authors.

The biomarkers included those commonly used in clinical practice (e.g., *EGFR*, Ki67, p53), as well as others selected based on recent scientific findings indicating their over-expression in malignant transformation. These included PD-L1, HLA-E, and B7-H6, which may also serve as potential molecular targets in immunotherapeutic approaches (Figure 1, Figure 2 and Figure 3). These figures are adapted from a previously published study [29], and are presented as illustrative examples of potential applications of OCB-based analysis for the detection of OC biomarkers. The original study was pilot study involving 15 patients.

Ki-67 is a nuclear protein expressed in all active phases of the cell cycle (G_1_, S, G_2_, and M) but absent in resting cells (G_0_), making it an established proliferation marker [47,48].

Gupta et al., 2023 [49] demonstrated that Ki-67 expression increases progressively from normal oral mucosa to oral epithelial dysplasia, reaching its highest level in OSCC. Higher Ki-67 expression corresponds to greater proliferative activity and lesion severity. OCB-based immunostaining for Ki-67 can therefore serve as a quantitative, reproducible indicator of epithelial proliferation during early oral carcinogenesis [50,51]

Regular follow-ups are essential for monitoring lesions at risk of malignant transformation, and specialised software can assist in tracking patient appointments and recording results [52]. When an OPMD lesion exhibits overexpression of specific tumour markers, it should be photographed for comparison and monitored through scheduled follow-up visits. It is recommended that immunological tests should be repeated at set time intervals, particularly if marker levels increase over time or if the number of positive markers rises, to detect early malignancy transformation [53].

Additionally, OCB sampling allows the harvesting of fresh tissue fragments through exfoliation. When combined with a highly sensitive ELISA, this approach may enable the detection of biomarkers that might otherwise go unnoticed. OCB sampling analysed using high-sensitivity ELISA has shown to distinguish tumour biomarker expression in OSCC from healthy tissue. These findings are consistent with results obtained using standard histology [29].

While immunohistochemistry provides valuable information, it often struggles to virtualise biomarkers at lower expression levels [54]. Immunological analysis of OCB samples has proven effective in distinguishing dysplastic lesions from a normal oral tissue. The method is rapid and can provide quantitative, reproducible results with high reliability and significant discriminatory value [29,55].

### 3.2. Epithelial Differentiation Marker

CKs are intermediate filament proteins that provide structural integrity to epithelial cells and reflect tissue-specific differentiation patterns. Alterations in CK expression occur during malignant transformation as epithelial cells lose differentiation and acquire invasive properties. Aberrant expression of CKs such as CK8, CK18, and CK19 has been observed in oral epithelial dysplasia and OSCC, and is associated with progression and invasive behaviour of the lesions [56]. These changes can be detectable via OCB-collected samples. They can serve as molecular indicators of dysplastic transformation before overt morphological changes become apparent [57]. The inclusion of CK profiling can enhance the sensitivity of immunological screening for early-stage oral lesions.

### 3.3. Growth Factor Signalling

*EGFR* is a transmembrane tyrosine kinase involved in regulating epithelial growth, survival, and differentiation. Aberrant activation of *EGFR* signalling promotes cellular proliferation, angiogenesis, and resistance to apoptosis, which are key features of tumour progression.

Overexpression of *EGFR* has been observed in OPMDs and OSCC with higher expression levels associated with dysplastic progression and tumourigenesis [58,59]. Immunohistochemical studies have demonstrated increased *EGFR* staining in oral dysplasia and OSCC compared to the normal mucosa, supporting its role as a biomarker of malignant transformation [58,59]. Moreover, non-invasive sampling of oral lesions using OCB can be combined with biomarker analysis such as high-sensitivity ELISA. This approach has been explored for detection of *EGFR*, which has been proposed as a potential biomarker associated with molecular alterations in dysplastic and neoplastic oral lesions [29].

### 3.4. Inflammatory Cytokines

Chronic inflammation contributes significantly to oral carcinogenesis through sustained oxidative stress, DNA damage, and creation of a tumour-promoting microenvironment. Several pro-inflammatory cytokines have been implicated as early biomarkers [60].

IL-1β is a master regulator of inflammatory signalling, promoting leukocyte recruitment, angiogenesis, and epithelial proliferation. Elevated IL-1β expression has been observed in oral epithelial dysplasia and OSCC tissues compared with healthy mucosa [38]. OCB-derived epithelial cells and saliva samples can provide reliable matrices for detecting IL-1β as an early immune-response biomarker [61].

IL-6 mediates tumour-promoting inflammation by activating JAK/STAT3 and MAPK signalling pathways, leading to enhanced cell survival and proliferation. Increased IL-6 levels are reported in precancerous oral lesions and early OSCC [62]. Measurement of IL-6 expression or secretion from OCB specimens may therefore serve as a sensitive early-detection parameter.

IL-8 acts as a chemokine that stimulates angiogenesis, cell migration, and tumour growth. Elevated IL-8 concentrations have been consistently observed in both unstimulated saliva and OCB samples from patients with early oral malignancies, distinguishing them from healthy controls [38]. IL-8 may serve as a high-specificity biomarker reflecting active tumour microenvironment remodelling.

TNF-α sustains chronic inflammation through NF-κB pathway activation, promoting the transcription of anti-apoptotic and proliferative genes. Elevated TNF-α levels correlate with disease progression from OPMDs to OSCC [62]. OCB-based immunoassays detecting TNF-α may aid in stratifying lesion risk during screening.

### 3.5. Immune Checkpoint/Emerging Markers

PD-L1 is a key immune checkpoint molecule expressed on tumour and epithelial cells that binds PD-1 receptors on T lymphocytes and natural killer (NK) cells, leading to immune suppression [39]. Its expression in early oral lesions suggests that immune evasion mechanisms are established during precancerous stages. Immunological detection of PD-L1 from OCB-derived cells may therefore help in identifying high-risk lesions with a higher likelihood of progression, and could inform future immunotherapy-based interventions [39].

B7-H6 (a ligand for the NK cell receptor NKp30) is emerging as a novel biomarker of tumour immune modulation. It is generally absent from most normal tissues but can be induced on tumour cells, where it engages NKp30 and contributes to innate immune recognition or modulation [63,64]. Expression of B7-H6 has been reported on various malignant tumour cell types. In some cases, its presence is associated with altered NKp30 expression and impaired NK cell function. This suggests that B7-H6 can play a role in tumour immune escape [63,64]. These features have led to interest in B7-H6 as a potential tumour immunomodulatory marker and therapeutic target.

Immunocytochemical or OCB-based analysis of B7-H6 and related immune-evasion markers may provide non-invasive insights into host–tumour interactions and support precision diagnostics in high-risk oral lesions [29].

## 4. Sample Collection and Immunological Analysis Methodology

Accurate immunological profiling of oral lesions depends on both the quality of the collected specimen and the robustness of downstream analytical methods. The OCB technique can offer a minimally invasive, reproducible, and patient-friendly approach for harvesting epithelial cells suitable for immunological and biomarker studies.

The following subsections describe the collection protocol, immunoassay strategies, and quality control considerations necessary to ensure precision and reproducibility in biomarker detection.

### 4.1. Cytobrush Collection Technique and Its Advantages

The OCB technique involves gently rotating a sterile nylon-tipped brush to collect squamous epithelial cells from the oral mucosa for cytological analysis [29]. The brush is typically applied with moderate pressure and rotated 5–10 times on the lesion site or the corresponding contralateral normal mucosa (for control samples). After collection, the OCB is placed into a sterile Eppendorf vial to ensure proper handling and preservation of the sample. Other systems also exist in which the OCB is placed directly into a transport medium such as phosphate-buffered saline (PBS), cytology fixative, or RNA preservation solution, depending on the downstream assay [38]. Additionally, a positive control is often used in confirmed cancer cases to validate the technique and ensure its accuracy.

Compared to traditional scalpel biopsy, OCB sampling offers several key advantages:

- Minimally invasive and painless: it eliminates the need for anaesthesia and reduces patient anxiety and post-procedural discomfort [65].
- Repeatable and site-specific: it allows for serial sampling of the same lesion or multiple mucosal sites for longitudinal monitoring [66].
- Reduced cost and time: suitable for outpatient and screening settings [67].
- Preserves cellular integrity: it provides intact epithelial cells and associated proteins for morphological and molecular analyses [55].
- Improves patient compliance [65].

Studies have confirmed the feasibility of using OCB-derived samples for immunocytochemistry, gene expression, and multiplex cytokine analysis, with comparable biomarker stability to biopsy-derived specimens [29,62]. The OCB approach also enables large-scale population screening, particularly in high-risk regions with low-resource settings or with limited access to histopathology diagnostic facilities.

In a separate case report by Rebaudi et al., 2024 [30], a 65-year-old male with proliferative verrucous leukoplakia (PVL), an OPMD, underwent both surgical biopsy and OCB sampling. Histopathology from the surgical biopsy confirmed a non-malignant lesion; however, immunological analysis of OCB-derived samples revealed overexpression of several biomarkers suggestive of a precancerous/dysplastic state. This finding highlights the potential of OCB for early detection of lesions at risk of progression to OSCC, allowing quantitative and qualitative assessments of biomarkers associated with malignant transformation. The study emphasised the importance of regular monitoring of high-risk lesions, with OCB sampling enabling longitudinal assessment of molecular changes before overt malignancy develops.

Additionally, it is important to highlight the potential use of the p53 protein and Ki-67 antigen as markers for malignant transformation and carcinogenesis in OPMDs and OSCC, respectively [34,68]. Elevated PD-1 and PD-L1 expressions in oral lesions indicate progression toward OSCC, while *EGFR* has been identified as both a prognostic and therapeutic target in OC [69].

### 4.2. Immunoassay Methods for Biomarker Detection

Immunological biomarker assessment from OCB specimens can be performed through several immunoassay platforms, depending on the target analyte and analytical purpose. These include the following:

#### 4.2.1. Immunocytochemistry (ICC)

The ICC enables localisation of protein biomarkers such as p53, Ki-67, and CKs directly within the epithelial cells collected via OCB. Cells are fixed on glass slides, incubated with primary antibodies, and visualised using chromogenic or fluorescent secondary detection systems. ICC provides semi-quantitative data on protein expression and subcellular localisation, facilitating correlation with histopathological features [29].

#### 4.2.2. Enzyme-Linked Immunosorbent Assay (ELISA)

ELISA is widely used for quantifying soluble cytokines and secreted proteins such as IL-6, IL-8, and TNF-α in OCB supernatants or lysates. The method is sensitive, cost-effective, and highly reproducible, enabling multiplexed quantification when combined with automated readers [38,62]. ELISA results can complement ICC data by providing quantitative immune signatures of inflammation or immune evasion.

#### 4.2.3. Immunofluorescence

Immunofluorescence and immunocytochemical assays provide high-resolution visualisation of biomarker localisation and cellular distribution using fluorophore-conjugated or enzyme-labelled antibodies. When applied to OCB-derived cytology cell block, these methods enable the detection of multiple targets, such as PD-L1, CKs, and proliferation markers, within the same sample, and facilitate digital image analysis for quantitative assessment of biomarker expression [70].

Immunocytochemistry on OCB samples has demonstrated feasibility and reliability. It enables antigen detection that is broadly comparable to tissue-based immunohistochemistry and may support its potential application in the early detection and surveillance of oral epithelial lesions [67].

#### 4.2.4. Multiplex Immunoassays

Recent advances in bead-based and planar-array multiplex immunoassays (e.g., Luminex^®^, MSD platforms, Luminex Corporation, Austin, TX, USA) enable simultaneous measurement of multiple cytokines, growth factors, and immune checkpoint proteins from small sample volumes [62,71]. These systems have been used to profile inflammatory biomarkers such as IL-1β, IL-6, IL-8, and TNF-α in OCB and salivary samples, demonstrating high sensitivity and throughput. Multiplex profiling may support a systems immunology approach to understanding early oral carcinogenesis.

### 4.3. Standardisation and Quality Control Considerations

For immunological analysis of OCB specimens to yield clinically reliable results, standardisation across sample collection, processing, and data interpretation is essential. Key considerations include:

- Pre-analytical variables such as the time from collection to processing, transport medium, and storage temperature can significantly affect the stability of proteins and cytokines. Immediate fixation or prompt processing is generally recommended, to preserve antigenicity for downstream assays. In some workflows, temporary storage at 4 °C prior to processing may slow biochemical degradation, but specific preservation methods (e.g., stabilising buffers, rapid fixation) should be chosen based on the target analyte and assay type [38].
- Sampling consistency: the number of brush rotations, the site selection (lesion vs. normal mucosa), and operator technique should be standardised and documented for reproducibility.
- Assay calibration: use of internal controls, duplicate testing, and calibration curves for each biomarker ensure quantitative reliability.
- Antibody validation: antibodies used for ICC, ELISA, or multiplex assays must be pre-validated for specificity and sensitivity in oral epithelial cells [29].
- Inter-laboratory harmonisation: cross-validation of results between laboratories or study centres helps establish standardised reference ranges for biomarker expression.

Implementing rigorous quality control procedures enhances the translational utility of OCB-based immunological assays, ensuring that results are comparable across clinical and research contexts. The development of standardised operating protocols for OCB collection and immunoassay execution is crucial for the integration of these tools into large-scale OSCC screening programmes.

## 5. Clinical Applications and Precision Medicine

The integration of immunological biomarkers derived from minimally invasive OCB samples into oral oncology has already begun to transform current screening and management paradigms. These approaches enable early cancer detection, as well as dynamic patient monitoring and individualised therapeutic decision-making, which are the key components of precision medicine [29,72,73]. Through the analysis of molecular changes in the oral mucosa, OCB sampling provides valuable insights into lesion behaviour and treatment efficacy, supporting personalised treatment strategies and facilitating more efficient clinical decision-making in real-world settings [29].

### 5.1. Biomarker-Based Risk Stratification and Early Intervention

Early detection remains the cornerstone of improving OSCC outcomes. Traditional visual and histological screening methods often fail to identify subclinical or molecularly active lesions. Immunological biomarkers provide a complementary, objective approach for risk stratification by detecting molecular changes before visible transformation occurs [37].

Biomarkers such as p53, Ki-67, and CKs indicate proliferative or dysplastic epithelial changes associated with early carcinogenesis. Inflammatory cytokines (IL-1β, IL-6, IL-8, and TNF-α) reflect a tumour-promoting microenvironment, whereas immune checkpoint molecules such as PD-L1 and B7-H6 indicate immune evasion [74]. When analysed together, these markers enable a multi-parametric risk model that classifies lesions as “low,” “intermediate,” and “high-risk” [39,75,76], as follows. Low-risk lesions: normal cytology, low Ki-67 index, and absence of inflammatory markers [74]; intermediate-risk lesions: elevated IL-6 and IL-8 with mild cytokeratin alterations [76]; and high-risk lesions: p53 overexpression, high Ki-67 index, strong PD-L1 positivity [40].

Such stratification enables targeted surveillance and early intervention. Hence, patients with high-risk molecular profiles can undergo closer follow-up or early excisional biopsy, while low-risk individuals can be monitored non-invasively, optimising healthcare resources and reducing overtreatment [74].

### 5.2. Monitoring Treatment Response

Immunological biomarkers from OCB specimens can also serve as dynamic indicators of treatment efficacy. During or after therapy, serial OCB sampling allows for real-time monitoring of cellular and molecular changes within the lesion microenvironment. A decrease in proliferation markers (Ki-67), normalisation of cytokine expression (IL-6, TNF-α), and downregulation of immune checkpoint molecules (PD-L1) may indicate favourable therapeutic response [38]. Conversely, persistently elevated biomarker levels could signal incomplete response, residual disease, or increased risk of recurrence.

As OCB sample collection can be painless and repeatable, it provides a practical alternative to repeated surgical biopsies, facilitating longitudinal monitoring without compromising patient comfort. Moreover, these data can be integrated with clinical and imaging findings to provide a comprehensive assessment of treatment outcomes. This approach supports adaptive therapy protocols that adjust treatment based on molecular response.

### 5.3. Integration into Precision Medicine Framework

The shift toward precision medicine in oncology emphasises tailoring diagnosis and therapy to individual molecular profiles, rather than relying solely on histopathological classification. In OSCC, OCB-derived biomarker profiling aligns directly with this framework by offering personalised and non-invasive molecular diagnostics [74]. Within a precision medicine model, immunological markers can serve multiple key roles [74]:

- Predictive: PD-L1 and B7-H6 expression may identify patients likely to benefit from immune checkpoint inhibitors.
- Prognostic: high levels of IL-6 or TNF-α may correlate with aggressive biological behaviour and poor survival.
- Diagnostic: elevated Ki-67 and p53 help confirm dysplastic transformation, even before morphological features become evident.

Integrating such markers with patient-specific data, including genetic background, lifestyle risk factors (tobacco, HPV status), and digital image analysis, can facilitate the development of AI-assisted predictive models for OC risk [77]. These models have the potential to refine patient management strategies and guide personalised prevention and intervention plans [77]. Future research will continue to optimise and expand OCB-based biomarker analysis. It may also integrate salivary proteomics and transcriptomics to create a comprehensive “liquid biopsy” platform for precision oral oncology [38].

### 5.4. Assessing Photobiomodulation Effects in OPMDs Using Biomarkers

Photobiomodulation (PBM) is a non-invasive therapeutic modality that uses low-level lasers or light-emitting diodes to modulate cellular and molecular activities, including energy metabolism (an increase in ATP production), signalling pathways, oxidative stress, and gene expression. These effects reduce inflammation, alleviate pain [78,79,80], and promote tissue repair [81,82]. Hanna et al., 2020 [81] demonstrated the potential role of PBM in managing oral mucosal lesions, including the OPMDs, with emphasis on erosive oral lichen planus (OLP) without dysplastic changes.

PBM has been shown to significantly improve clinical lesion severity and patient symptoms in OLP. At the molecular level, PBM therapy has reduced salivary inflammatory cytokines (IL-1β, IL-6, TNF-α) in patients with OLP [81], and increased expression of anti-apoptotic (bcl-2) and proliferative (Ki-67) markers in lesion tissue, suggesting recovery of cell cycle mechanisms and enhanced keratinocyte proliferation [83]. These findings highlight the fact that PBM exerts measurable effects at both clinical and molecular levels in non-dysplastic OLP lesions and support the use of serial biomarker profiling to objectively monitor tissue responses to therapy.

## 6. Clinical Protocol for Performing OCB

**Figure 4:**
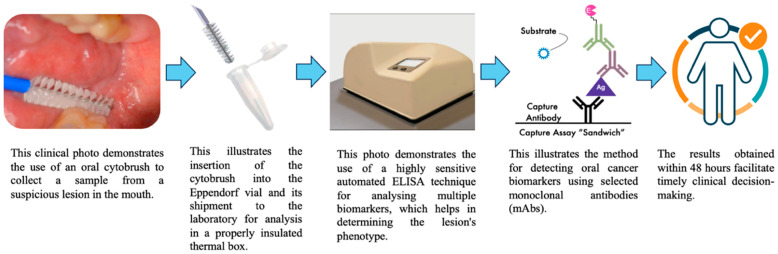
Schematic illustrating the workflow from the dental chair to point-of-care analysis. Processing of the OCB sample requires only a few simple steps, and enables the early detection of OC biomarkers. Early identification of these biomarkers supports improved survival outcomes and monitoring of OPMDs. The figure is an original schematic created by the authors.

The step-by-step OCB execution process for obtaining cell samples for diagnostic purposes and cell characterisation is suitable for molecular and immunological analysis. Figure 4 illustrates the steps from the dental chair, through detecting tumour biomarkers to achieving a better prognosis.

**Figure 5:**
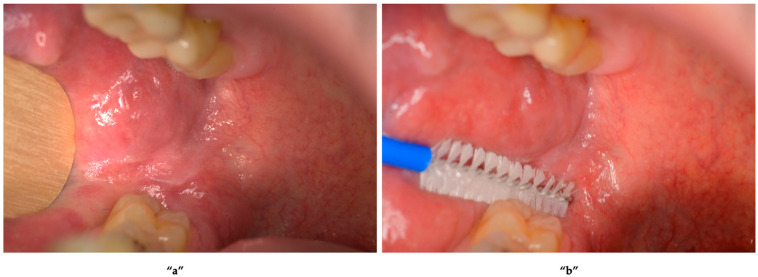
Clinical photo illustrating a suspicious oral mucosal lesion and the oral cytobrush (OCB) technique for specimen collection. **“a”** OCB performed on a suspicious lesion in the right retromolar pad area; **“b”** the sampling technique, involving gentle rubbing of the OCB over the lesion to collect a tissue specimen, followed by rotating the brush, to ensure an adequate specimen for analysis. Image courtesy of the authors; provided for illustrative purposes only.

Based on scientific literature and the authors’ collective experiences, we have outlined a step-by-step guide to how clinicians can effectively utilise the OCB in a clinical setting:

- Initial patient consultation: a thorough history should be taken from the patient with a suspicious oral mucosal lesion, identifying associated risk factors for OSCC [84], such as smoking, alcohol consumption, poor oral hygiene, a family history of cancer, previous HPV infection, autoimmune or dermatological diseases, or typical symptoms of mouth or throat cancer.
- Extraoral examination: detecting any lymph nodes involvement is as important as the for identifying any oral abnormalities or any changes in the oral mucosa (Figure 5a).

- Documentation of the findings: all the findings should be documented in the patient’s records, including initial intraoral photos of the lesion, location (mucosal, submucosal, and intraosseous), colour, number, duration, size, shape, borders, surface texture, consistency on palpation, and any visible or palpable vascular pulsations.
- Chair-side OCB screening: perform OCB screening to collect cyto-salivary samples from the suspected area for tumour immunological biomarker analysis, aiming to detect any early malignant transformation [28,29].
- Informed consent: prior to performing the OCB procedure, obtain written consent from the patient after providing a full explanation of the test procedure, its benefits and potential post-operative complications.
- Pre-sampling procedure: before OCB sampling, the patient should rinse their mouth with only sterile normal saline solution to help remove any superficial debris and excess saliva. Oral disinfectants or antiseptic mouthwashes (e.g., chlorhexidine) should not be used immediately prior to sampling, as they can alter cellular morphology. Moreover, there is currently no evidence in the literature supporting an improvement in cytology specimen quality when they are used.

The sterile water rinse is intended to improve cell yield and specimen quality for both cytological and molecular analyses. Formal removal of necrotic tissue is not routinely recommended, as the literature does not provide standardised guidance for this step in OCB sampling.

**Figure 6:**
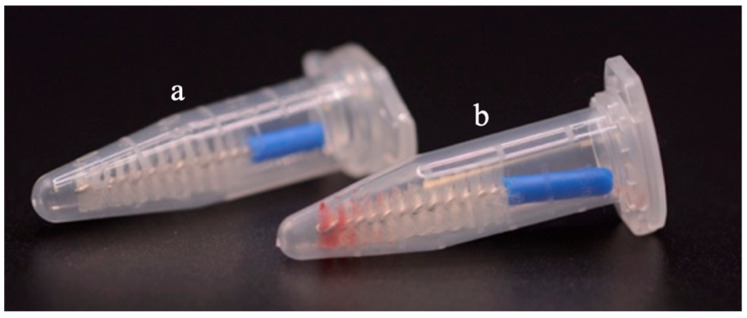
An illustrative image showing proper versus unsuitable OCB sample collection. (**a**): OCB collected following correct procedure without contamination; (**b**) OCB sample n deemed unsuitable for analysis, due to significant blood contamination. Image courtesy of the authors.

- OCB sampling: the OCB should be utilised by vigorously brushing over the entire lesion, with a motion that combines both brushing and rotation, to ensure a sufficient number of cells are collected for analysis (Figure 5b).
- Appropriate sample collection: the OCB should not be contaminated by blood, pyrogens or disinfectants. After sampling, the OCB should be placed into an Eppendorf tube, and the excess handle should be cut off using shears. Figure 6a shows an OCB following a proper cell collection without visible contamination, such as blood, pyrogens, or disinfectants, whereas the OCB in Figure 6b was deemed unsuitable for analysis, due to significant blood contamination.

- Sample storage and laboratory delivery: place the vial inside a thermal box with a frozen ice pack, accompanied by a detailed report.
- Follow-up appointment: schedule a follow-up appointment one week after the test to inform the patient of the diagnosis and, subsequently, plan the necessary treatment.

## 7. Challenges and Limitations

Despite promising advancements in immunological biomarker research and OCB-based diagnostics, several challenges must be addressed before routine clinical translation is feasible. These include inter-patient variability, methodological standardisation, and the need for large-scale validation.

### 7.1. Standardisation of Collection and Assay Protocols

Lack of standardisation in OCB collection methods and immunoassay protocols remains a significant barrier to reproducibility across studies [38].

Variations in sampling technique (pressure applied, number of rotations, and lesion site), fixation protocols, antibody selection, and detection platforms can lead to inconsistent biomarker measurements [37].

Developing SOPs and implementing quality control checkpoints, such as using reference controls, defined OCB pressure, and validated antibody panels will be essential for generating comparable and clinically interpretable data.

Harmonisation across laboratories and clinical centres can also facilitate the creation of international biomarker reference databases that improve cross-study validation.

Furthermore, ensuring appropriate storage and transport conditions for OCB specimens (e.g., maintaining cold chain, avoiding repeated freeze–thaw cycles) is critical for preserving antigen integrity and avoiding false-negative or false-positive findings [85].

### 7.2. Need for Large-Scale Validation Studies

While numerous small-scale studies have demonstrated the feasibility of detecting immunological biomarkers from OCB samples, large, multicentre validation studies are required to confirm diagnostic accuracy and clinical utility. Most current data are derived from small cohorts, often lacking diverse demographic and geographic representation [37]. Future validation efforts should include:

- Prospective longitudinal designs to evaluate biomarker performance over time in predicting malignant transformation [86].
- Multivariate analyses combining immunological, histological, and genetic data for robust risk modelling [87].
- Comparison with gold-standard biopsy and histopathology to define sensitivity, specificity, and predictive values [88].

Such studies are essential to translate OCB-based immunological profiling from research settings into standardised clinical workflows and to obtain regulatory approval for diagnostic use [29].

## 8. Future Directions

The convergence of advanced technologies, such as AI, multi-omics, and integrated diagnostic platforms, offers new opportunities to enhance the precision and predictive power of immunological analysis for OSCC.

### 8.1. AI and Multi-Omics Integration

AI and machine learning algorithms can analyse complex datasets generated from immunoassays, genomics, transcriptomics, and proteomics. Integrating multi-omics data from OCB specimens allows the identification of novel biomarker patterns predictive of early malignant transformation [89].

For example, integrating immunological profiles (IL-6, PD-L1 expression) with transcriptomic signatures of epithelial–mesenchymal transition and other molecular features can improve classification and biomarker discovery in OSCC through bioinformatic analysis [90,91]. In addition, AI and assisted-machine learning workflows have been developed for automated analysis of immunocytochemistry or immunofluorescence images. These approaches can enhance diagnostic precision, reduce observer bias, and enable quantitative assessment of cellular and molecular markers in OSCC [92,93]. These computational frameworks can eventually evolve into clinical decision-support systems, guiding clinicians toward personalised treatment planning.

### 8.2. Development of Biomarker Panels for Improved Sensitivity and Specificity

Single biomarkers often lack the diagnostic power to distinguish between inflammation and neoplastic transformation. Therefore, the development of multi-marker panels that combine tumour suppressor, proliferation, and immune checkpoint proteins with cytokine signatures can have the potential to significantly enhance diagnostic precision [94].

Panels that include p53, Ki-67, IL-6, IL-8, TNF-α, and PD-L1 have already shown promising sensitivity (>85%) and specificity (>80%) in pilot studies using OCB samples [38]. Future studies should focus on optimising these panels for high-throughput, point-of-care immunoassays that are compatible with community-level screening [94].

### 8.3. Combination of Immunological Analysis with Salivary Diagnostics

Saliva, being rich in soluble proteins and extracellular vesicles, represents a complementary diagnostic medium to OCB samples. Integrating OCB-based cellular biomarkers with salivary and serum immunological and proteomic data could yield a comprehensive, non-invasive diagnostic platform [85,95].

For instance, salivary cytokines such as IL-1β, IL-6, and IL-8 can be measured alongside OCB-expressed markers, including p53, PD-L1, CKs. This dual-layered diagnostic system can capture both cellular and secretory alterations associated with early carcinogenesis [38,62]. Such multimodal approaches align closely with the principles of precision prevention and early molecular surveillance in oral oncology.

## 9. Conclusions

Immunological analysis of cytobrush-collected specimens represents a transformative advance for precision medicine in OSCC care. Among the biomarkers reviewed, panels combining dysregulated tumour suppressors and proliferation markers (e.g., p53 and Ki-67) with epithelial differentiation markers (CK13 and CK17) show the strongest evidence for detecting early dysplasia. Similarly, inflammatory cytokine panels (IL-6, IL-8, TNF-α) and immune checkpoint molecules (PD-L1, B7-H6) are promising for assessing early malignant transformation and immune evasion. These biomarker combinations are closest to clinical translation, as they offer reproducible detection from minimally invasive, repeatable cytobrush specimens. Incorporating these panels into precision medicine strategies can improve prognostic accuracy, enable risk stratification, and guide patient-specific surveillance and therapeutic decisions. Future integration with multi-omics and salivary diagnostics may further enhance the predictive and diagnostic utility of OCB-based immunological screening, establishing it as a cornerstone of next-generation OSCC prevention and management.

## Figures and Tables

**Table 1:** Representative biomarkers analysed using OCB and related specimens, summarising their biological pathways and clinical relevance in oral epithelial dysplasia and OSCC. Quantitative diagnostic performance (e.g., sensitivity and specificity) is not consistently reported across studies. The table is intended as a qualitative overview of biomarker relevance.

Biomarker	Specimen Type	Pathway/Role	Clinical Relevance	Reference
p53	Cytobrush, epithelial cells, tissue	Tumour suppressor; DNA damage response; apoptosis	Early dysplasia detection; correlates with progression to OSCC	[34]
Ki-67	Cytobrush cells, tissue	Proliferation marker; cell cycle regulation	Indicates proliferative activity; higher index in dysplasia and OSCC	[35]
Cytokeratins (CK17, CK13)	Cytobrush cells, tissue	Epithelial differentiation	Distinguishes dysplastic vs. normal epithelium; aberrant expression in early lesions	[36]
IL-6	Cytobrush, unstimulated saliva	JAK/STAT3 pathway; pro-inflammatory	Promotes proliferation, angiogenesis, elevated in OSCC	[37,39]
IL-8	Cytobrush, unstimulated saliva	Chemokine neutrophil recruitment, angiogenesis	Elevated in dysplasia and OSCC, sensitive early marker	[37]
IL-1β	Cytobrush, unstimulated saliva	Pro-inflammatory cytokine: NF-κB pathway	Early inflammatory changes, marker of tumour-promoting microenvironment	[38]
TNF-α	Cytobrush, unstimulated saliva	NF-κB activation: pro-inflammatory	Sustains tumour-promoting inflammation; high diagnostic accuracy	[38]
PD-L1	Cytobrush, tissue	Immune checkpoint; T-cell inhibition	Indicates immune evasion; potential therapeutic target	[39]
B7-H6	Cytobrush, tissue	Immune checkpoint; NK cell modulation	Tumour-restricted expression; early immune evasion marker	[40]

## Data Availability

All the data are available in the text.
